# Mining and characterization of the PKS–NRPS hybrid for epicoccamide A: a mannosylated tetramate derivative from *Epicoccum* sp. CPCC 400996

**DOI:** 10.1186/s12934-022-01975-2

**Published:** 2022-11-24

**Authors:** Tao Zhang, Guowei Cai, Xiaoting Rong, Jingwen Xu, Bingya Jiang, Hao Wang, Xinxin Li, Lu Wang, Ran Zhang, Wenni He, Liyan Yu

**Affiliations:** 1 Institute of Medicinal Biotechnology, Chinese Academy of Medical Sciences & Peking Union Medical College, Beijing, 100050 China; 2 Medical Research Center, Binzhou Medical University Hospital, Binzhou, 256603 Shandong China; 3 College of Biotechnology, Jiangsu University of Science and Technology, Zhenjiang, 212003 Jiangsu China

**Keywords:** Epicoccamide, Genome mining, PKS−NRPS hybrid, Tetramates, Biosynthesis, *Epicoccum*

## Abstract

**Background:**

Genomic analysis indicated that the genomes of ascomycetes might carry dozens of biosynthetic gene clusters (BGCs), yet many clusters have remained enigmatic. The ascomycete genus *Epicoccum*, belonging to the family Didymellaceae, is ubiquitous that colonizes different types of substrates and is associated with phyllosphere or decaying vegetation. Species of this genus are prolific producers of bioactive substances. The epicoccamides, as biosynthetically distinct mannosylated tetramate, were first isolated in 2003 from *Epicoccum* sp. In this study, using a combination of genome mining, chemical identification, genetic deletion, and bioinformatic analysis, we identified the required BGC *epi* responsible for epicoccamide A biosynthesis in *Epicoccum* sp. CPCC 400996.

**Results:**

The unconventional biosynthetic gene cluster *epi* was obtained from an endophyte *Epicoccum* sp. CPCC 400996 through AntiSMASH-based genome mining. The cluster *epi* includes six putative open reading frames (*epi*A-*epi*F) altogether, in which the *epiA* encodes a tetramate-forming polyketide synthase and nonribosomal peptide synthetases (PKS−NRPS hybrid). Sequence alignments and bioinformatic analysis to other metabolic pathways of fungal tetramates, we proposed that the gene cluster *epi* could be involved in generating epicoccamides. Genetic knockout of *epiA* completely abolished the biosynthesis of epicoccamide A (**1**), thereby establishing the correlation between the BGC *epi* and biosynthesis of epicoccamide A. Bioinformatic adenylation domain signature analysis of EpiA and other fungal PKS-NRPSs (NRPs) indicated that the EpiA is l-alanine incorporating tetramates megasynthase. Furthermore, based on the molecular structures of epicoccamide A and deduced gene functions of the cluster *epi*, a hypothetic metabolic pathway for biosynthesizing compound **1** was proposed. The corresponding tetramates releasing during epicoccamide A biosynthesis was catalyzed through Dieckmann-type cyclization, in which the reductive (R) domain residing in terminal module of EpiA accomplished the conversion. These results unveiled the underlying mechanism of epicoccamides biosynthesis and these findings might provide opportunities for derivatization of epicoccamides or generation of new chemical entities.

**Conclusion:**

Genome mining and genetic inactivation experiments unveiled a previously uncharacterized PKS − NRPS hybrid-based BGC *epi* responsible for the generation of epicoccamide A (**1**) in endophyte *Epicoccum* sp. CPCC 400996. In addition, based on the gene cluster data, a hypothetical biosynthetic pathway of epicoccamide A was proposed.

**Supplementary Information:**

The online version contains supplementary material available at 10.1186/s12934-022-01975-2.

## Background

Filamentous fungi have been demonstrated to be attractive and rich repertoires for pharmaceutical drugs in human history [1, 2]. However, it is becoming challenging to acquire novel lead compounds with traditional chemical-only methodologies for high frequency of rediscovery of known molecules [3]. Thus, several approaches have been employed to activate cryptic biosynthetic pathways, including “OSMAC” approach [4, 5], co-culture [6, 7], and chemical epigenetic agents supplement [8, 9]. Additionally, genome sequencing has revolutionized natural products (NPs) exploitation endeavor, demonstrating that the capacity of filamentous fungi to yield compounds was far more than we anticipated [2, 10]. Meanwhile, we could now easily access to the sequenced genome of targeted microorganism whose metabolites have not been, or rarely, characterized. Genome-guided mining approach is usually conducted to set up the linking between secondary metabolites identification and targeted biosynthetic gene clusters (BGCs) verification [11, 12]. Also, previous studies have demonstrated that genome mining is an effective approach that facilitated exploitation for new metabolites with fascinating structures, and numerous BGCs were investigated [10, 13, 14]. Thus, the genome-guided metabolomic analysis strategy renders the bioprospecting of bioactive compounds effective and promising.

**Fig. 1:**
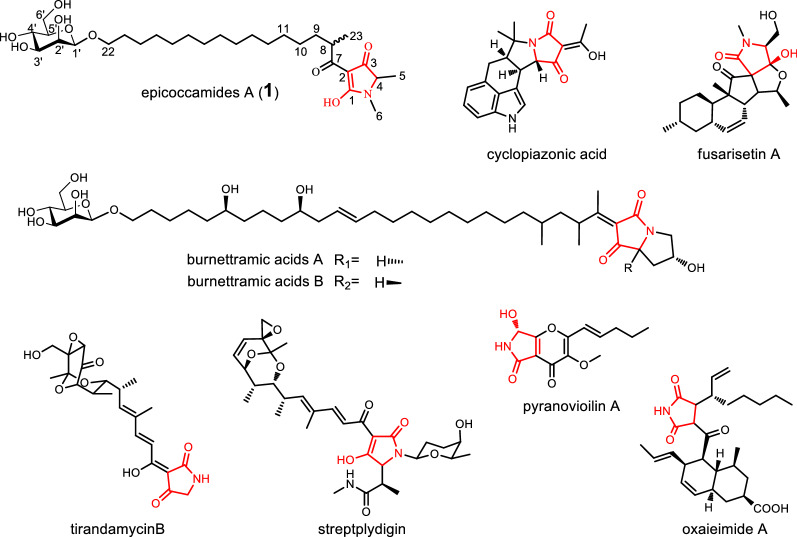
Representatives of naturally occurring tetramic acid derivatives. The pyrrolidine-2, 4-dione ring unit is in red

Tetramic acid derivatives bearing pyrrolidine-2, 4-dione ring system, comprise a structurally unique family of secondary metabolites (SMs) (Fig. 1) [15]. Naturally occurring tetramates have attracted increasing interest from pharmaceutical and research communities due to their diverse bioactivities including antibacterial, fungicidal, antiviral, and cytotoxic potencies [16–18]. Biosynthetically, most tetramate derivatives are originated from polyketide synthase-nonribosomal peptide synthase (PKS−NRPSs) hybrid pathways, and these polyketide −peptide compounds are generated from Dieckmann condensation of an amino acid (or nonproteinogenic) to a polyketide-derived acyl chain [16, 19]. Recently, postgenomic analysis accelerated the biosynthetic studies on this class of SMs, and numerous tetramate-forming biocatalysts have been elucidated, as exemplified by aspyridone [20], tenellin [21], tirandamycin [15], cyclopiazonic acid [22], fusarisetin A [23], pseurotin [24], streptolydigin [25], pyranoviolin [26], oxaleimide A [27], burnettramic acid [28], and calipyridone [29]. Although these studies revealed the molecular bases for these tetramates and demonstrated that diverse arrays of fascinating biocatalysts are the key components for their structural diversification [16, 18]. However, many biosynthetic reactions still remain to be unveiled.

*Epicoccum* genus is distributed widespread, which has been frequently isolated as endophytes residing in medicinal plants [30] and found to live in association with other organism [31, 32]. Species from this genus are prolific producers of bioactive secondary metabolites, including epicorazines, (dithio)diketopiperazines, triornicin, flavipins, epicocconigrones, epicoccolides, epicocconone, and epicolactone dimers (beetleane A, epicoane A) [14, 31, 33–35]. The metabolites isolated from *Epicoccum* strains, cover a diverse array of bioactivities including strong inhibitors of liver fibrosis, cytotoxic and antimicrobial activities, making them potential drug candidates [31]. The epicoccamides, as biosynthetically distinct tetramic acid glycoside, were first isolated in 2003 from a jellyfish-derived culture of *E*. *purpurascens* and later identified in *Epicoccum* sp. [36] However, the genetic basis of this metabolite has been elusive and needs to be investigated. Recently, Li and coworkers characterized a gene cluster *bua* involving in the burnettramic acid generation from *Aspergillus burnettii*, which revealed that the PKS−NRPS hybrid BuaA and the mannoyltransferase BuaB functioned in the formation of proline-incorporating polyketide aglycone skeleton and β-D-mannose transferring, respectively [28]. This example strongly proved that genome-guided metabolite mining is a feasible approach to mine potential secondary metabolites [28]. Therefore, the exploitation for rare or new *Epicoccum* species contributed to develop an efficient strategy for targeted genome mining of tetramates and discovery of new bioactive compounds.

Herein, we investigated an uncharacterized tetramate-forming gene cluster in the fungal strain *Epicoccum* sp. CPCC 400996. Furthermore, one mannosylated pyrrolizidinedione molecule named epicoccamide A was readily identified. Intriguingly, using a combination of genetic knockout, bioinformatic analysis, and a plausible biosynthetic pathway of **1** was also proposed. Our study has implications for developing a strategy for rapid targeted linkage of secondary metabolites to their encoding BGCs by genome mining and facilitates future applications for generation of bioactive molecules through metabolic engineering.

## Materials and methods 

### Strains, plasmids, and culture

The fungus *Epicoccum* sp. CPCC 400996 was isolated from the medicinal plant, deposited in the China Pharmaceutical Culture Collection Center (CPCC), and used as the parental strain. The strain was routinely cultured on potato dextrose medium (PDA; BD company) under conditions at 28 °C. *Escherichia coli* DH5α (GenScript) was used for DNA molecular cloning. *Agrobacterium tumefaciens* strain AGL-1 was employed as helper strain during *A*. *tumefacien*s-mediated transformation of fungal *Epicoccum*. The induction medium (IM) utilized for co-culture experiments was prepared as described elsewhere [37]. The strains, plasmids, primers, and derived mutants are listed in Table 1 and preserved in 15% glycerol at − 80 °C. Large scale fermentation of *Epicoccum* cultures were performed using rice medium (80 g/100 mL, autoclaved at 121 °C for 30 min) and cultivated at 28 °C for 30 days.

**Table 1:** Strains, plasmids and oligonucleotides used in this study

Strains	Description	Source
*E. coli* Trans T1	*lac*х*74 recA1 deoR F – mcrA* Δ (*mrr-hsdRMS-mcrBC*) *ϕ80 lacZ*Δ*M15*Δ *araD139*Δ *(ara-leu)7697 galU galK*	Transgen
T1-Δ*epiA*	*E* .*coli* cell carrying disruption cassette Δ*epiA*	This study
*Agrobacetrium tumefaciens* (AGL-1)	Carrying pCAMBIA3301 plasmid, Kan^R^	[42]
AGL-1-Δ*epiA*	AGL-1 cell carrying Δ*epiA*	This study
*Epicoccum* sp. CPCC 400996	Wild type strain	
Δ *epiA* -5	*epiA* deletant of CPCC 400996	This study
Δ *epiA* -11	*epiA* deletant of CPCC 400996	This study

Plasmids	Description	Source
pAg1-H3	Vector for targeted gene replacement containing HYG gene as selection marker	[59]
H3-*epiA*-Dn	3′ prime arms of *epiA* gene cloned into pAg1-H3	This study
H3-Δ*epiA*	5′ & 3′ prime arms of *epiA* gene cloned into pAg1-H3	This study

Oligonucleotides	Sequence (5′–3′)	Purpose
Z01 (*epiA*-Up-F)	CGACTAGTTTCATGTCAGCATGTAGC	5′ prime arm amplication
Z02 (*epiA*-Up-R)	CATTAATTAATGAGTCACGAACCTATT	
Z03 (*epiA*-Dn-F)	ATCTCGAGTCAAACGCCAAAATGAAC	3′ prime arm amplication
Z04 (*epiA*-Dn-R)	ATGGGCCCTGAGCCCACCCGTGCCGG	
Z05 (*epiA*_In-F)	ATTGATGAAGGAGAAGAGGGAG	In deletion region characterization
Z06 (*epiA*_In-R)	GGAAACCAGGTTCGCAGT	
Z07 (cha-*epiA*-UF)	GGGGCTACAATCTTTACG	*epiA* 5′ prime characterization
Z08 (cha-*epiA*-UR)	GATACCAGACAACCACCTT	
Z09 (cha-*epiA*-DF)	GTAAGCGGCACCAATCTC	*epiA* 3′ prime characterization
Z10 (cha-*epiA*-DR)	AGCAACATCCCATCACAA	
Z11 (cha-hph-F)	GGGCAAAGGAATAGAGTAGATG	Hyg + gene characterization
Z12 (cha-hph-R)	TGCAATAGGTCAGGCTCT	

### Sequencing and bioinformatic analysis

The genomic DNA (gDNA) of strain *Epicoccum* sp. CPCC 400996 was extracted using CTAB extraction buffer denoted previously [37]. The gDNA was recovered followed by chlorform extraction, precipitation with isopropanol and further washing with 75% alcohol. The precipitated gDNA was dissolved in 1 mL deionized water, digested with RNase, and purified by chloroform extraction. Then the precipitated gDNA was dried and redissolved in 300 μL deionized water. The shotgun genome sequencing was performed at Shanghai Majorbio Bio-pharm Technology Co. (Shanghai, China) on an Illumina Hiseq 2000 system. Sequence assembly was performed with SOAP denovo 1.05 [38]. Gene annotations were then conducted with GenomeMatcher [39] for local BLAST searching and analyzed via FGENESH platform (www.softberry.com). Protein sequences were compared with the BlastP program in the NCBI database. AntiSMASH database was carried out for bioinformatic mining of metabolic clusters [40]. PKS/NRPS analysis online platform was used for in silico prediction of PKS/NRPS domain organization [41].

### General techniques for DNA manipulation

For constructing the disruption cassette of the gene *epiA*, the homologous arms of the *epiA* was amplified from gDNA of strain CPCC 400996 using primer sets (*epiA*-Up-F/R and *epiA*-Dn-F/R, respectively) listed in Table 1. PCR amplifications were carried out on a Veriti 96 Well Thermal Cycler (Applied Biosystems) using PrimeSTAR^®^GXL DNA polymerase (Takara). The two PCR amplicons were ligated into a pEASY^®^-Blunt vector (Transgen) and confirmed through DNA sequencing. The downstream amplicon *epiA*-Dn was recovered by restriction enzymes digestion using *Xho*I and *Apa*I (New England Biolabs), then ligated into simultaneously linearized with *Xho*I/*Apa*I digested pAg1-H3 vector to create the plasmid H3-*epiA*-Dn (Table 1). In addition, the upstream amplicon *epiA*-Up was recovered by *Spe*I/*Pac*I (New England Biolabs) double enzyme digestions, and then ligated into linearized vector H3-*epiA*-Dn to create the plasmid H3-Δ*epiA*. The H3-Δ*epiA* was transformed into *A*. *tumefacien*s cells. DNA restriction endonucleases were employed following the instructions as recommended by the manufacturer. Primers employed to amplify the genes are listed in Table 1.

### Generating *Epicoccum*-derived deletant *ΔepiA*

The gene deletion of *epiA* was performed by *A*. *tumefacien*s-mediated target gene disruption system, as previously described for *Pseudogymnoascus dustructans* [42]. The deletant strain was obtained by replacing targeted gene sequence with the selective hygromycin marker in wild type strain of CPCC 400996. The *A*. *tumefacien*s-mediated transformation (ATMT) of *Epicoccum* sp. was performed with minor modifications. The *Agrobacterium* cells were transformed with disruption plasmid H3-Δ*epiA* by electroporation. The co-cultivation experiments were conducted at 30 °C for 72 h. The randomly selected transformants were then inoculated onto selective plates comprising of cefotaxime (250 µg/mL) and hygromycin (150 µg/mL). The mutants were transferred into PDB selection broth to verify the genotype by diagnostic PCRs following the extraction of gDNA. Primers used for diagnositc PCR verification are listed in Table 1. The comparison in amplicons size between the target gene replaced by the hygromycin selection marker and the native gene promoted us to verify whether the mutants exhibited the correct gene deletion.

### Chemicals and chemical analysis

High performance liquid chromatography (HPLC) analyses were conducted on an Agilent 1290 system with an Agilent-SBC18 column (4.6 × 250 mm, 5 μm). Thin Layer Chromatography (TLC) was carried out on silica gel GF254 plates from Qingdao marine chemical company, China. The medium pressure liquid chromatography was performed on Combi Flash Rf 200 (Teledyne Isco, Lincoln NE, USA) with a SEPAF FLASH^®^ Flash silica gel column (330 g, Santai Technologies, China). Nuclear Magnetic Resonance (NMR) data was acquired using Bruker AVIII-600 spectrometer (150 MHz for ^13^C NMR and 600 MHz for ^1^H NMR, Bruker Corporation, Karlsruhe, Germany). High resolution electrospray ionization mass spectra (HRESIMS) were recorded on a Thermo LTQ Orbitrap XL Mass Spectrometer (Thermo Fisher Scientific, USA).

### Scale-up production, isolation of metabolite epicoccamide A

Seed cultures of the strain CPCC 400996 were cultivated in 500 ml flasks (×5) each containing 200 mL of liquid medium (PDB, BD company) at 30 °C on a rotary shaker (220 rpm). After 96 h, 5 mL of the seed culture was used to inoculate into 500 mL erlenmeyer flask (×50) with a rice fermentation medium (rice 100 g/flask, deionized H_2_O 100 mL/flask). After 30 days cultivation, the cultures were harvested, extracted 2 times with methanol (3 × 30 L) for 30 min each under an ultrasonic bath (10% power intensity). The total methanol solutions were concentrated under reduced pressure to afford the dried extract (60.5 g). Then the methanol extract was dispersed in aqueous (1 L) and extracted by using petroleum ether (3 × 1 L) and ethyl acetate (3 × 1 L) successively. The ethyl acetate residue (5.7 g) was subjected to silica gel column chromatography eluting with a petroleum ether and ethyl acetate mixed solvent system in a step gradient (100:0–0:100, v/v) to afford five fractions (Fr.1–Fr.5). Fr.4 (5.4 g) was further chromatographed on Sephdex LH20 and ODS-MPLC (RP-C18) subsequently. The purification of sub-fractions was carried out on semi preparative HPLC [YMC-Pack ODS-A, 10 mm × 250 mm, 5 μm, flow rate: 3.5 mL/min, 55% acetonitrile-water (v/v)] to obtain epicoccamide A (**1**) (9.8 mg, *tR* 14.0 min).

## Results

### Bioinformatic analysis of the biosynthetic gene cluster *epi*

**Fig. 2:**
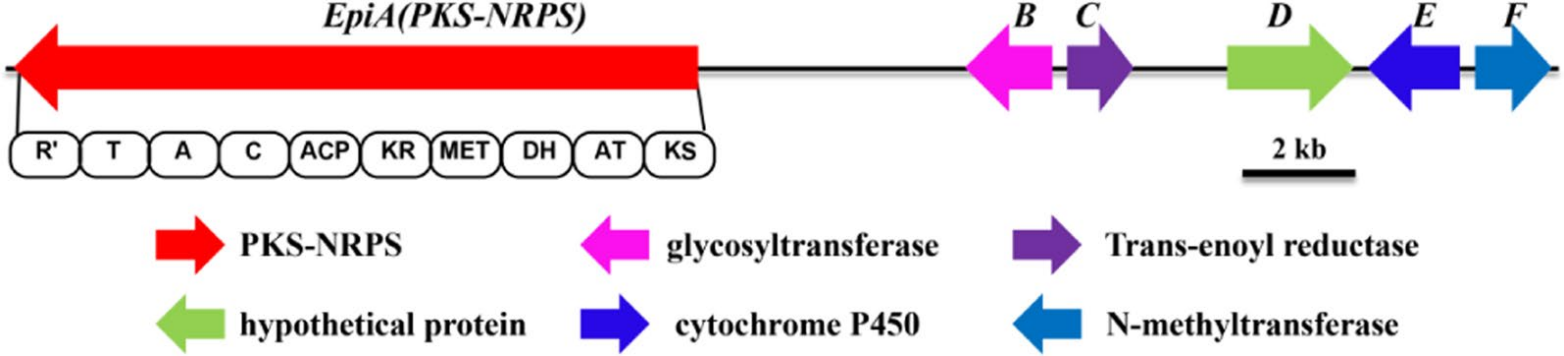
Genetic organization of the epicoccamide A biosynthetic locus found in the *Epicoccum* sp. CPCC 400996 genome. The PKS − NRPS module abbreviations are: KS, ketosynthase; AT, malonyl-CoA transferase; MET, methyltransferase; DH, dehydratase; KR, ketoreductase; ER, enoyl reductase; ACP, acyl carrier protein; SAM, S-adenosyl-l-methionine; C, condensation domain; A, adenylation domain; T, thiolation domain; R, reductive domain. Predicted functions of individual ORFs are summarized in Table 2

We utilized a genome sequencing strategy to investigate the biosynthetic potential of the fungus *Epicoccum* sp. CPCC 400996. The Illumina HiSeq 2500 sequencing of the strain produced ~ 4148 million bases in total. Assembly of the unpaired shotgun sequencing reads promoted us to obtain 514 contigs, which includes 30.92 million nonredundant bases. After careful bioinformatic analyses of the genome with AntiSMASH platform (https://fungismash.secondarymetabolites.org), we obtained two biosynthetic gene clusters in the genome of the strain CPCC 400996 that carry glycosyltransferase encoding gene. Of particular, one gene cluster caught our attention, which was somewhat similar to the BGC of the burnettramic acid and was designated as the *epi* cluster (Fig. 2; Table 2). Six putative open reading frames (*epi*A–*epi*F) altogether were acquired based on bioinformatic analysis of the continuous DNA region. The protein EpiA has the domain organization of KS-AT-DH-MET-KR-ACP-C-A-T-R as verified by in silico analysis. BlastP alignment illustrated that the EpiA showed 53% identity to hybrid PKS−NRPS synthetase BuaA in *A*. *burnettii*, followed by the fusaridione A synthetase FsdS, fusarisetin A synthetase Fsa1, equisetin synthetase EqxS, pyrrolocin synthetase PrlS, pyrichalasin H synthase PyiS, and Ilicicolin H IccA, members of fungal tetramate-forming PKS−NRPS hybrids.

**Table 2:** Putative functions of ORFs within the BGC *epi* involved in epicoccamide A biosynthesis

Protein	Bua homologues (coverage/identity)	Eqx homologues (coverage/identity)	Putative functions
EpiA	BuaA (97%/53%)	EqxS (86%/38%)	PKS-NRPS hybrid
EpiB	BuaB (89%/40%)	ND	Glycosyltransferase
EpiC	BuaC (95%/57%)	EqxC (92%/43%)	Trans-enoyl reductase
EpiD	ND	ND	hypothetical protein
EpiE	BuaG (93%/48%)	ND	Cytochrome P450
EpiF	ND	EqxD (98%/48%)	N-methyltransferase

Further closer investigation on the bilaterial sequences of the *epiA* facilitated the discovery of the auxilliary genes encoding tailoring enzymes for tetramic acid late stage modification. The gene products conclude one glycosyltransferase (EpiB), one LovC-like trans-enoyl reductase (EpiC), one cytochrome P450 monooxygenase (EpiE), one methyltransferase (EpiF), and one hypothetical protein (EpiD) that are in accord with the structural features of glycosylated tetramic acid derivatives. Although the biosynthetic gene cluster *epi* is homologous to the *bua* in *A*. *burnettii*, the majority of coding gene products exhibit > 50% sequence identical [28]. Nevertheless, compared to the biosynthetic gene cluster *bua*, the *epi* lacks two genes that code cytochrome P450 (*buaD*) and proline hydroxylase (*buaE*), but instead one more methyltransferase coding *epiC* was discovered. Additionally, BlastP alignment indicated that EpiF exhibited 47–49% identities to EqxD, Fsa4, and PynC, respectively, and the three methyltransferases are believed to catalyze the methyl group incorporation at N position during biosynthesis of tetramates equisetin [43], fusarisetin [27], and pyranonigrin [44]. Since tetramate glycoside epicoccamides have been isolated from two *Epicoccum* strains, we speculated that the fungus could produce epicoccamides and the gene cluster *epi* might be able to responsible for the biosynthesis of these molecules.

### Structure elucidation of epicoccamide A from *Epicoccum* sp. CPCC 400996

Compound **1** was purified as yellow oil, which had a molecular formula of C_29_H_51_NO_9_ as verified by ESIMS (*m/z* 556.76, calculated for C_29_H_51_NO_9_ [M-H]^−^). On the basis of ^1^H, ^13^C NMR and HMQC data, along with the analysis of literature data [36], Compound **1** was suggested as an epicoccamide derivative—epicoccamide A. The NMR data were shown as follows, in ^1^H NMR data (in DMSO, 600 MHz): *δ*_H_ 3.87 (1H, br.s, H-4), 1.23 (3H, d, *J* = 6.3 Hz, H-5), 2.88 (3H, s, H-6), 3.50 (1H, m, H-8), 1.58 (1H, m, H-9), 1.41 (1H, m, H-9), 1.20–1.29 (m, H-10–H-20), 1.50 (2H, m, H-21), 3.39(1H, m, H-22), 3.74(1H, m, H-22), 1.08 (3H, d, *J* = 6.7Hz, H-23), 4.33 (1H, br.d, H-1′), 3.60 (1H, m, H-2′), 3.23 (1H, m, H-3′), 3.29 (1H, m, H-4′), 3.00 (1H, m, H-5′), 3.45 (1H, m, H-6′) and 3.67 (1H, m, H-6′). The ^13^C NMR data (in DMSO, 151 MHz): *δ*_C_ 172.3 (C-1), 100.2 (C-2), 189.8 (C-3), 62.2 (C-4), 14.4 (C-5), 26.0 (C-6), 194.5 (C-7), 35.2 (C-8), 33.0 (C-9), 26.6 (C-10), 28.8-29.2-(C-11-C-19), 25.6 (C-20), 29.2 (C-21), 68.4 (C-22), 16.9 (C-23), 100.2 (C-1′), 70.6 (C-2′), 73.7 (C-3′), 67.2 (C-4′), 77.5 (C-5′) and 61.4 (C-6′), which were similar to epicoccamide derivatives (Additional file 1: Figs. S1-S2). According to the above analyses, compound **1** was composed of three moieties as a tetramic acid, an aliphatic chain, and a hexose. The structures of the latter two moieties were verified by ^1^H-^1^H COSY, HMBC and ESI MS (Additional file 1: Table S1, Figs. S3–S6) analysis. Furthermore, the teramide acid moiety was determined by HMBC correlations from H-5 (*δ*_H_ 1.23) to C-3 (*δ*_C_ 189.8) and C-4 (*δ*_C_ 62.2), and from H-6 (*δ*_H_ 2.88) to C-1 (*δ*_C_ 172.3) and C-4 (*δ*_C_ 62.2), coupled with ESI MS/MS (−) data (Additional file 1: Fig. S7) at *m/z* 126.08 for the molecular fragment resulting from the *α*-cleavage of the teramide acid moiety. Thus, compound **1** was identified as epicoccamide A and the structure was shown in Fig. 1.

### Characterization of the BGC *epi *responsible for the yielding of epicoccamide A

To verify the participation of EpiA in the yielding of epicoccamide A, disruption of the PKS−NRPS megasynthase coding gene *epiA* was conducted. We performed a gene inactivation experiment to supply the evidence that *epiA* is responsible for epicoccamide A biosynthesis. Prior to ATMT experiment, we tested two antibiotics including hygromycin B and G418. Hygromycin concentration of 100 µg/mL could completely inhibit the growth of the parental strain and was used as following selective antibiotic. A selective cassette HygR (pTrpC-HygR-tTrpC) consisting of the promoter coding gene *ptrpC*, hygromycin resistance gene (HygR), and the terminator coding gene *tTrpC* was constructed by conventional restriction enzymes digestion and ligation strategy. The knockout plasmid construction was performed based on the scaffold of pAg1-H3 by replacing one of the two nearly continuous fragments from the region of the gene *epiA* on bilateral of the HygR selective cassette.

**Fig. 3:**
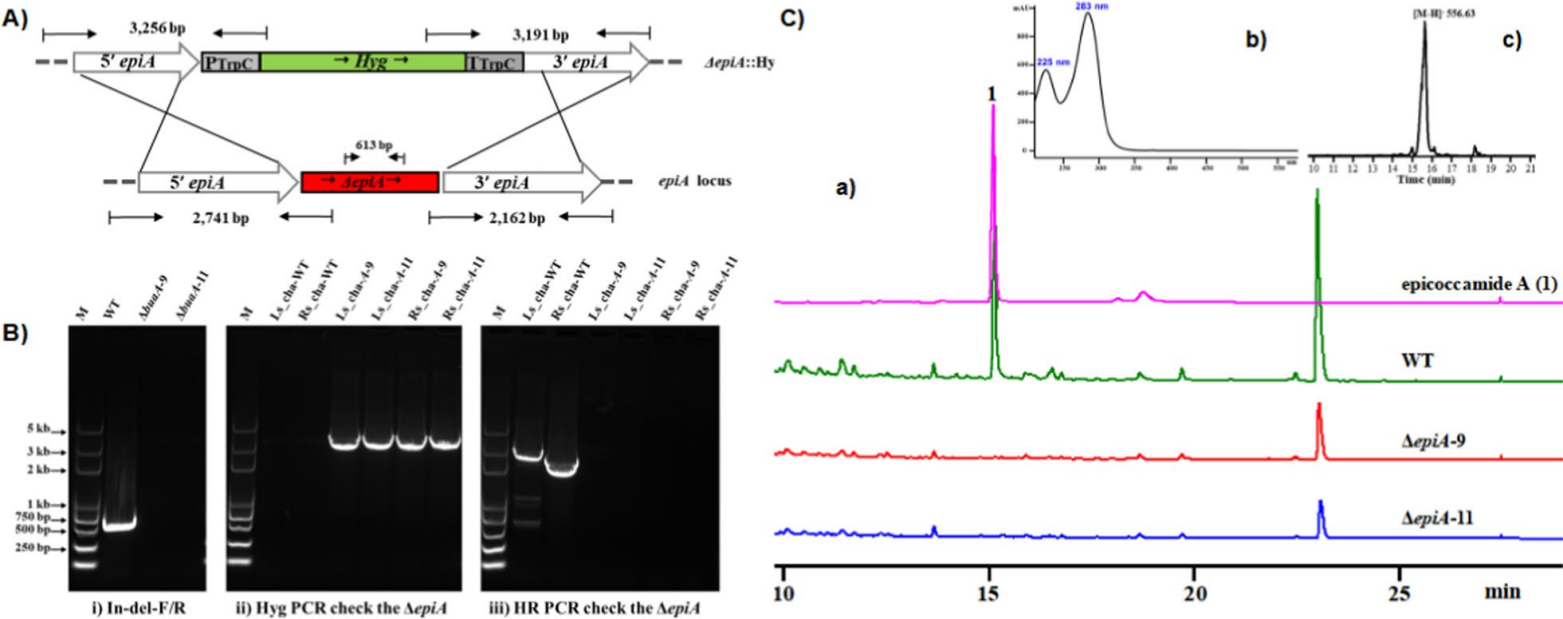
Gene deletion of *epiA* and characterization of Δ*epiA* mutants. **A** Schematic representation of the *epiA*−disruption cassette with the genetic map and its surrounding regions. The dashed line represents expected size product for wild-type and deletant strains. **B** Diagnostic PCR characterization of Δ*epiA* mutants. Primer pair (Z05/Z06) located within the deletion region of *epiA* gene (Bi-613 bp in size for WT and no PCR amplicon for mutants); Primer pairs (Z07/Z12 and Z10/Z11) targeting the *Hyg* gene and *epiA* flanking regions (Bii-3256 bp and 3191 bp in size for mutants and no PCR amplicon for WT); Primer pairs (Z07/Z08 and Z09/Z10) located in the *epiA* deletion region and flanking regions (Biii-2741 bp and 2162 bp in size for WT and no PCR amplicon for mutants). **C** HPLC profiles of organic extracts obtained from the WT and Δ*epiA* mutants of *Epicoccum* sp. The positions of epicoccamide A are indicated in the chromatograms. The elution was monitored at 280 nm and shown on the same Y-scale. HPLC-MS analysis of Δ*epiA* metabolites in positive ion mode, EIC at *m/z* 556, [M-H]^−^.

Next, after *A*. *tumefacien*s-mediated transformation experiment, of 19 putative Hyg^+^ *Epicoccum* transformants analyzed, the mutants including Δ*epiA*-9 and Δ*epiA*-11 were verified by diagnostic PCR characterization. The primer pairs resided within the deleted region of the *epiA*, yielding a 613-bp PCR amplicon for wild type (WT) strain but not for the selected mutants (Fig. 3A, Bi). The mutants that had no amplicons were further characterized for Δ*epiA*::HygB^+^ integration at the homologous site by using four additional primers pairs. With one *HygR* specific primer set, both mutants had two amplicons of 3256 bp and 3191 bp in size, respectively, while the control WT gave no amplicons (Fig. 3A, Bii). Further verification with one primer set located in deletion region of *epiA*, two amplicons with 2741 bp and 2162 bp in size were amplified from WT, whereas no amplicons were recovered from both mutants (Fig. 3A, Biii). The thus-obtained crude extracts from Δ*epiA*-9 and Δ*epiA*-11, and parental strain were analyzed for its metabolites profile by high performance liquid chromatography (HPLC). At the same HPLC condition, the peak of epicoccamide A at 10 min that confirmed it is the target compound along with the evidence of *m/z* 556 [M-H]^−^. However, as shown in Fig. 3C, the mutant strains Δ*epiA*-9 and Δ*epiA*-11 completely abolished the production of **1**. These results further corroborated with our prediction regarding the function of the gene cluster *epi*, confirming that the gene *epiA* is responsible for epicoccamide A biosynthesis in *Epicoccum* sp.

### Investigation on the biosynthesis of metabolite epicoccamide A

**Fig. 4:**
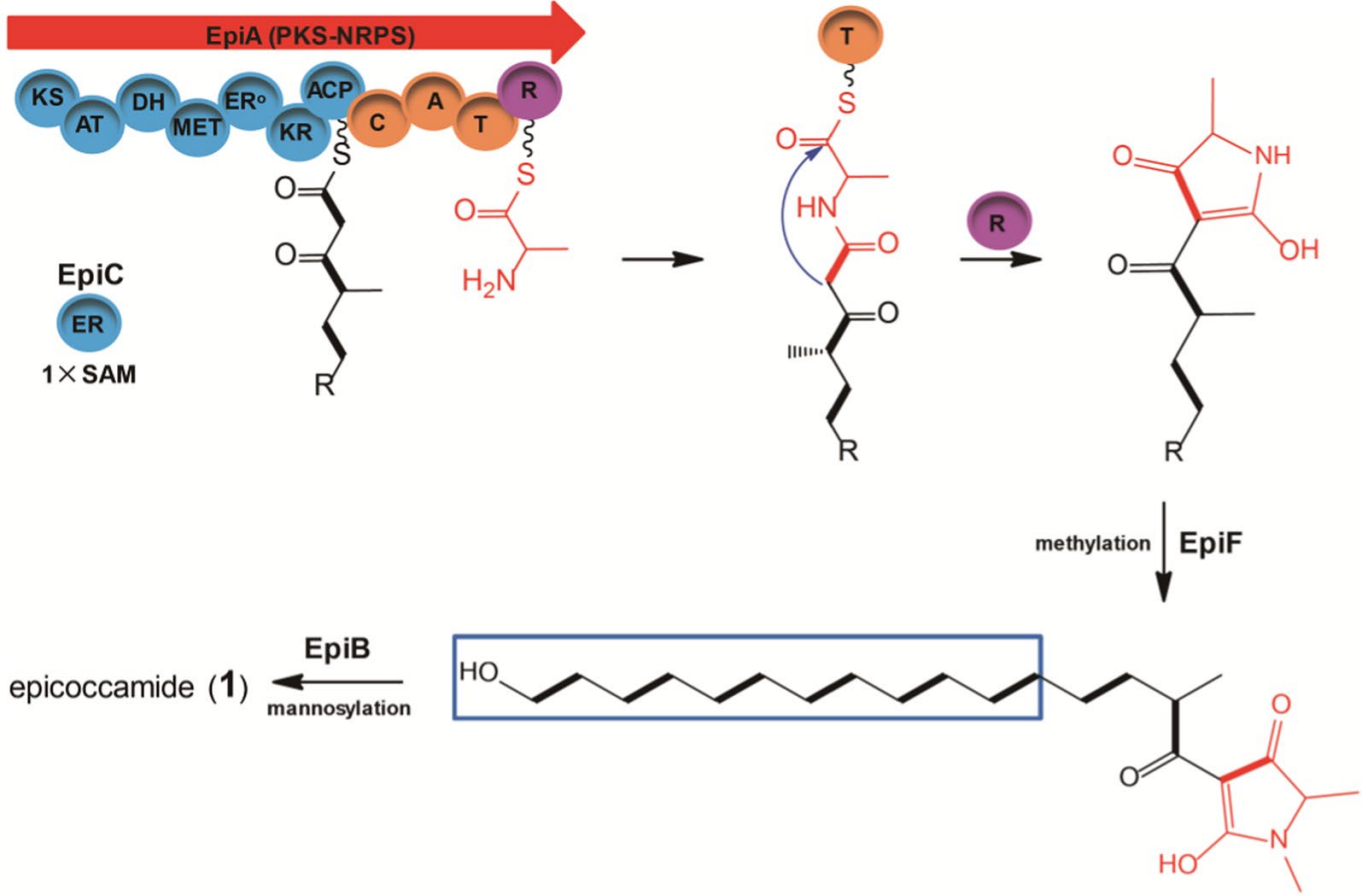
Domain organization and proposed biosynthetic pathway of epicoccamide A (**1**). SAM, S-adenosylmethionine. Modules in blue denote incorporation of the starter unit from malonyl-CoA or methylmalonyl-CoA, and those in orange denote incorporation of L-alanine

Epicoccamides are unique glycosylated tetramic acid derivatives featuring a linear aliphatic chain attached to tetramate and mannose moieties. The genetic base for the biosynthesis of burnettramic acid was well determined previously, and the biosynthesis of the epicoccamide A should be highly similar to that of burnettramic acid [34]. In the light of the molecular structures of epicoccamide A and proposed gene functions of the gene cluster *epi*, a biosynthetic pathway of epicoccamide A was envisioned (Fig. 4). Domains of the megasynthase EpiA are programmed and generate polyketide chains with varied methylation and reduction modifications and NRPS modules are in charge of fusing the polyketide chain to an l-alanine and an offloading R domain for tetramate cyclization and chain release. Of this compound, acetate is extended seven times with seven unities of malonate by PKS domains, programmed C-methylation occurs following the first two extensions derived from an intact methylmalonate precursor, and cycles of full reduction occur after the first two extensions. The following steps involve the methyltransferase EpiF for installation of the methyl group into the nitrogen position of tetramate ring and glycosyltransferase EpiB to accomplish the β-d-mannose transferring reaction. However, in the burnettramic acid pathway, the polyketide chain of intermediate burnettramic acid aglycones occur multiple hydroxylations oxygenated by the cytochrome P450 BuaG, followed by BuaB-catalyzed mannosyltransferring for the generation of final product burnettramic acid. In contrast, the intermediate epicoccamide aglycone was N-methylated to afford the N-methyl modified intermediates, a feature that is lacking in burnettramic acid. Additionally, it is important to note that cytochrome P450 EpiE exhibited higher identities (> 40%) to P450s including HimC, BuaG, and AscH, which participated in hydroxylations or multiple oxidations of the polyketide chain during himeic acid A, and burnettramic acid biosynthesis, respectively [34, 45]. We believed that novel and further oxygenated derivatives of epicoccamides catalyzed by P450 EpiE would be mined from *Epicoccum* sp.

**Fig. 5:**
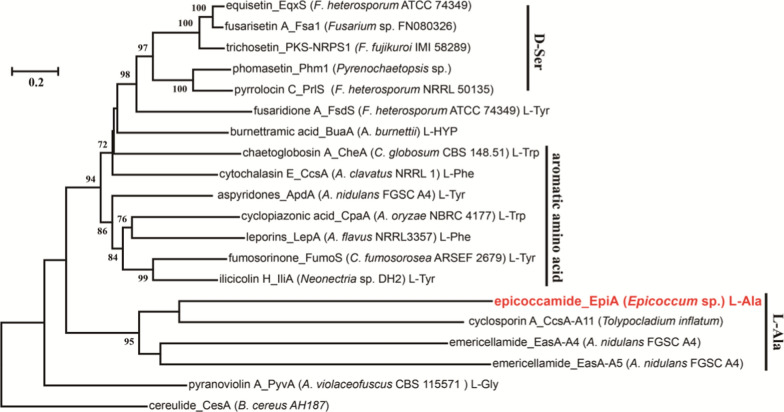
Phylogenetic tree of fungal adenylation domain signature sequences retrieved from characterized multimodular PKS−NRPS hybrid synthase or peptide synthetases (NRPSs). The EpiA was highlighted in red. The scale represents changes per site; numbers at branches are bootstrap values. For accession numbers and further details, the reader is referred to the Additional file 1: Table S2

EpiA is quite an unconventional fungal PKS − NRPS megasynthase, as it is only the first fungal PKS−NRPS that has been exhibited to specifically recognize and activate the amino acid of l-alanine. We next phylogenetically analyzed the adenylation domains of characterized PKS-NRPSs from biosynthetic pathways of fungal tetramates. Similar analyses were performed on embedded adenylation domains in NRPSs for cyclic peptides including cyclosporin A and emericellamides [46, 47]. Notably, in this phylogenetic dendrogram, EpiA, EasA-A4, and EasA-A5 clustered in a distinct branch with CcsA-A11 of cyclosporin A formation in *Tolypocladium inflatum*, which are more inclined to acquire L-alanine as precursors (Fig. 5). Additionally, the 10 signature residues for the adenylation domains of the alanine-incorporating EpiA indicated that positions 235, 236, and 517 are conserved (D_235_L_236_K_517_), while others are flexible (Additional file 1: Table S1). We believe their significance for alanine might be uncovered with increasing alanine-incorporating PKS-NRPSs.

## Discussion

Using a targeted gene deletion, we have identified the *epi* family genes encoding the biosynthetic pathway for the tetramate derivative epicoccamide A in the fungal strain CPCC 400996. Interestingly, during our research on epicoccamides biosynthesis, Kwon and coworkers also characterized the genetic BGC (*epc*) of epicoccamide in *E*. *nigrum* KACC 40,642 [48]. It is worth noting that both *epi* and *epc* gene clusters are syntenic and highly identical, and this further demonstrated our proposal that the cluster *epi* is responsible for biosynthesizing epicoccamides [48]. Since the study of the tetramaic acid-originated epicoccamides identified from *E*. *purpurascens* two decades ago, the underlying genetic base and biochemcial transformation of this class of compounds remain to be characterized [36, 46]. With genome sequences becoming accessible due to plummeting costs, genomics-based methods are now commonplace and might hold the key to lead a renaissance in the field of secondary metabolites discovery [4, 13, 49, 50]. The understanding to link secondary metabolites to their corresponding BGCs and vice versa, along with ever-increasing understanding of biosynthetic machineries, has inspired the field of genome mining-guided secondary metabolites exploitation for the rational discovery of new chemical entities [3, 51]. In this report, genome guided bioinformatic analysis of the fungus CPCC 400996 revealed the localization of a novel PKS-NRPS hybrid biosynthetic gene cluster. Additionally, the gene cluster *epi* also includes one glycosyltransferase encoding gene *epiB* and one N-methyltransferase encoding gene *epiF*, which implies that this gene cluster might be the candidate for epicoccamides biosynthesis in the fungus *Epicoccum* sp. CPCC 400996 (Fig. 2). Taken together, a genome-guided secondary metabolites mining approach was performed to discover the compound epicoccamide A in the producer of *Epicoccum* sp. strain CPCC 400996.

**Fig. 6:**
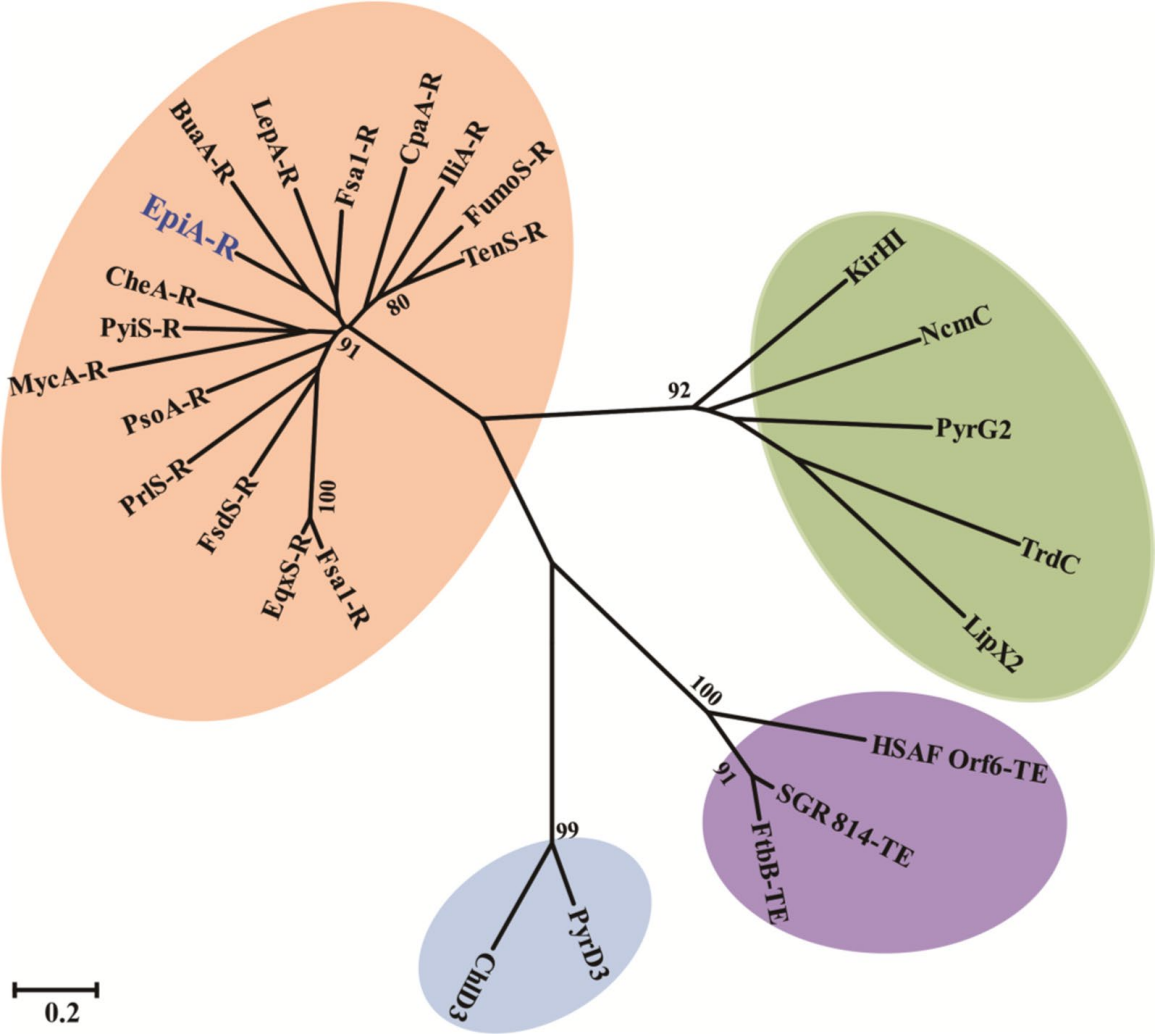
Unrooted phylogenetic tree of NRPS R domains or proteins catalyzing Dieckmann cyclization during tetramates biosynthesis. NRPS R domains involved in fungal tetramates metabolic pathways; TrdC family cyclases involved in actinomycete-derived tetramates; TEs involved in bacterial polycyclic tetramic macrolactams (PTMs) biosynthesis; PyrD3, and ChlD3 involved in pyrrolidine-2,4-dione ring formation during pyrroindomycin and chlorothricin synthesis, respectively

We next phylogenetically analyzed the EpiA R domain with the NRPS R or R^’^ domains from fungal metabolic pathways and the corresponding Dieckmann cyclases or TE domains involved in bacteria-derived tetramates [15]. Four paradigms have been proposed to decipher the releasing mechanism of tetramate derivatives. These conclude the following conditions: (i) the action of a hybrid PKS-NRPS megasynthase TE carrying module involved in bacterial polycyclic tetramic macrolactams (PTMs) biosynthesis, exemplified as the HSAF [52], FtbB, and SGR-814, for which the terminal TE module of Orf6 in the HSAF biosynthetic pathway (carrying peptide ligase and protease activities) catalyzes tetramate generation [52]; (ii) the action of a reductive (R) domain locating in terminal module within the fungal PKS-NRPS hybrids as highlighted by tenellin [17], equisetin [16], fusaridione A [53], and burnettramic acid [28], in which the dissected R domain of FsdS has been demonstrated in vitro to catalyze Dieckmann-type cyclization for releasing corresponding tetramates; (iii) the action of Dieckmann cyclases as reflected by the actinomycete-derived metabolites including tirandamycin[15], kirromycin [54], and α-lipomycin [55], in which TrdC family cyclases could in vitro catalyze the cyclization of tetramate scaffolds; and (iv) chemistry performed in generating bacteria-derived spiro-linked tetramates including pyrroindomycins [56] and chlorothricins [57], these include a phylogenetically unique two gene ensemble PyrD3/PyrD4 and ChlD3/ChlD4. In this phylogenetic tree, EpiA R domain clustered in a separate branch including NRPS R or R^’^ domains involved in fungal tetramates biosynthesis (Fig. 6). This suggested that it might support the proposal that the releasing mechanism or cyclizations are conserved and unique during biosynthesis of fungal tetramates.

**Fig. 7:**
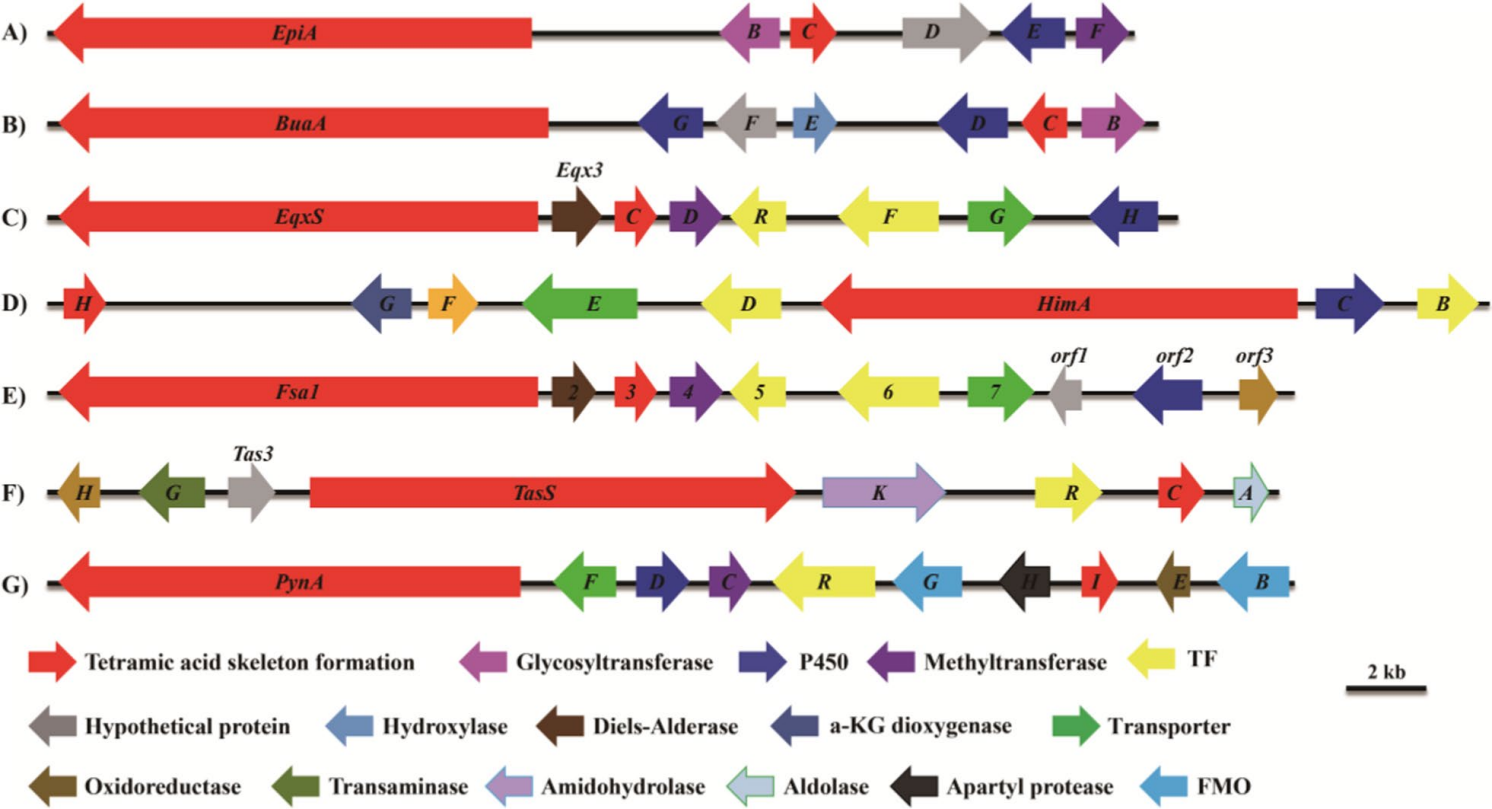
Evolutionary conserved biosynthetic gene clusters of fungal tetramates: **A** epicoccamide A from *Epicoccum* sp. CPCC 400996; **B** burnettramic acids from *Aspergillus burnettii* FRR 5400; **C** equisetin from *Fusarium heterosporum* ATCC 74349; **D** himeic acid from *A*. *japonicus* MF275; **E** fusarisetin A from *Fusarium* sp. FN080326; **F** Sch210971/2 from *Hapsidospora irregularis*; **G** pyranoviolin A from *A*. *violaceofuscus* CBS 115571

Investigation of the biosynhtetic gene cluster *epi* revealed neighboring genes that are highly homologous to reported *bua* cluster in *A*. *burnettii* genome. These two gene clusters contained a deduced 5-membered heterocycle formation encoding genes together with other putative tailoring ORFs conserved among the previously reported biosynthetic pathways of tetramate derivatives (Fig. 7). A more detailed cluster blast bioinformatic analysis was carried out on other fungal tetramates including equisetin from *Fusarium heterosporum* ATCC 74,349 [14], himeic acid from *Aspergillus japonicus* MF275 [13], fusarisetin A from *Fusarium* sp. FN080326 [23], Sch210971/2 from *Hapsidospora irregularis* BP-2511 [58], and pyranoviolin A from *A*. *violaceofuscus* CBS 115,571 [26]. Significantly, all these TETases genes are related to a double-gene ensemble that catalyzes the formation of the tetramate moiety. The ensemble includes genes that encode a PKS-NRPS hybrid and a trans-enoyl reductase (ER). The prevalence of these TETases-encoding gene clusters across these fungi has promoted the conclusion that they might encode for yielding of a group of structurally related tetramic acids that have a common metabolic origin (Fig. 7). Comparisons of these fungal tetramates encoding gene clusters will contribute us to elucidate the biosynthetic mechanisms of this unique class of secondary metabolites. The genetic investigations of these fascinating biocatalysts will facilitate us to rationally generate novel epicoccamide derivatives with structural diversification and improve pharmacological property.

## Concluding remarks

In conclusion, we identified a previously uncharacterized PKS − NRPS hybrid based biosynthetic gene cluster of epicoccamide A (**1**) from *Epicoccum* sp. CPCC 400996, which enables us to investigate the genetic mechanism of epicoccamide A biosynthesis. The gene cluster *epi* for generating the compound (**1**) was verified by gene inactivation experiment in the native producer. Based on the gene cluster data, a putative metabolic pathway of epicoccamide A was proposed. These findings might provide the feasibility of using them for further investigation of epicoccamides biosynthesis. Our study demonstrates that genome mining targeted strategy would accelerate the discovery of secondary metabolites, and also provide opportunities for derivatization of epicoccamides via manipulating biosynthetic pathway.

## Supplementary Information

**Additional file 1**: **Table S1.** NMR Data for epicoccamide A (**1**). **Table S2.** Comparison of the signature residues for amino acid selection by adenylation (A) domains of fungal PKS-NRPS (NRPS). **Figure S1.** The ^1^H NMR spectrum of compound **1**. **Figure S2.** The ^13^C NMR spectrum of compound **1**. **Figure S3.** The ^1^H-^1^H COSY spectrum of compound **1** (in DMSO). **Figure S4.** The HSQC spectrum of compound **1** (in DMSO). **Figure S5.** The HMBC spectrum of compound **1** (in DMSO). **Figure S6.** HRESIMS spectrum of compound **1**. **Figure S7.** ESI MS/MS (-) spectrum of compound **1**.

## Data Availability

All data generated during this study are included in this article, and all material is available upon request.
