# Supplementary material for: Mining and characterization of the PKS–NRPS hybrid for epicoccamide A: a mannosylated tetramate derivative from Epicoccum sp. CPCC 400996
Source: Microb Cell Fact. 2022 Nov 24;21:249. doi: 10.1186/s12934-022-01975-2 (PMC9685919; doi:10.1186/s12934-022-01975-2)
Supplement: Supplementary file 1 — Additional file 1: Table S1. NMR Data for epicoccamide A (1). Table S2. Comparison of the signature residues for amino acid selection by adenylation (A) domains of fungal PKS-NRPS (NRPS). Figure S1. The 1H NMR spectrum of compound 1. Figure S2. The 13C NMR spectrum of compound 1. Figure S3. The 1H-1H COSY spectrum of compound 1 (in DMSO). Figure S4. The HSQC spectrum of compound 1 (in DMSO). Figure S5. The HMBC spectrum of compound 1 (in DMSO). Figure S6. HRESIMS spectrum of compound 1. Figure S7. ESI MS/MS (-) spectrum of compound 1. [file 12934_2022_1975_MOESM1_ESM.docx]

Additional file 1

Mining and Characterization of the PKS−NRPS Hybrid for Epicoccamide A: a Mannosylated Tetramate Derivative from *Epicoccum* sp. CPCC 400996

Tao Zhang^a*^, Guowei Cai^a,b^, Xiaoting Rong^a,c^, Jingwen Xu^a^, Bingya Jiang^a^, Hao Wang^a^, Xinxin Li^a^, Lu Wang^a^, Ran Zhang^a^, Wenni He^a*^, Liyan Yu^a*^

^a^ *Institute of Medicinal Biotechnology, Chinese Academy of Medical Sciences & Peking Union Medical College, Beijing 100050, China*

^b^ *Medical research center, Binzhou medical university hospital, Binzhou, Shandong province, China, 256603*

^c^ *College of Biotechnology, Jiangsu University of Science and Technology, Zhenjiang, Jiangsu, China, 212003*

Corresponding Authors

Tao Zhang - *Institute of Medicinal Biotechnology*, *Chinese Academy of Medical Sciences & Peking Union Medical College, Beijing 100050, China;* Email: evergo218@163.com

Wenni He - *Institute of Medicinal Biotechnology*, *Chinese Academy of Medical Sciences & Peking Union Medical College, Beijing 100050, China;* Email: [wnner0047@126.com](mailto:zhangt218@hotmail.com)

Liyan Yu - *Institute of Medicinal Biotechnology*, *Chinese Academy of Medical Sciences & Peking Union Medical College, Beijing 100050, China;* Email: yly@cpcc.ac.cn.

**CONTENTS**

| **Contents** | **Page** |
| --- | --- |
| Table S1. NMR Data for epicoccamide A (**1**) | S3 |
| Table S2. Comparison of the signature residues for amino acid selection by adenylation (A) domains of fungal PKS-NRPS (NRPS) | S4-5 |
| Figure S1. The ^1^H NMR spectrum of compound **1** | S6 |
| Figure S2. The ^13^C NMR spectrum of compound **1** | S7 |
| Figure S3. The ^1^H-^1^H COSY spectrum of compound **1** (in DMSO) | S8 |
| Figure S4. The HSQC spectrum of compound **1** (in DMSO) | S9 |
| Figure S5. The HMBC spectrum of compound **1** (in DMSO) | S10 |
| Figure S6. HRESIMS spectrum of compound **1** | S11 |
| Figure S7. ESI MS/MS (-) spectrum of compound **1** | S12 |

**Table S1. NMR Data for epicoccamide A (1).**

| Position | *δ*_C_ | *δ*_H_ (*J* in Hz) | ^1^H-^1^H COSY | HMBC |
| --- | --- | --- | --- | --- |
| 1 | 172.3 |  |  |  |
| 2 | 100.2 |  |  |  |
| 3 | 189.8 |  |  |  |
| 4 | 62.2 | 3.87, br. s | H-5 |  |
| 5 | 14.4 | 1.23, d (6.3) | H-4 | C-3, C-4 |
| 6 | 26.0 | 2.88, s |  | C-1, C-4 |
| 7 | 194.5 |  |  |  |
| 8 | 35.2 | 3.50, br. s | H-9, H-23 |  |
| 9 | 33.0 | 1.41, m , 1.58, m | H-8, H-10 | C-10, C-11 |
| 10 | 26.6 | 1.20~1.29  overlapped |  |  |
| 11-19 | 28.8**-**29.2 |  |  |  |
| 20 | 25.6 |  |  |  |
| 21 | 29.2 | 1.50, m | H-22 | C-19, C-20, C-22 |
| 22 | 68.4 | 3.39, m, 3.74, m | H-21 | C-20, C-21, C-1' |
| 23 | 16.9 | 1.08, d (6.7) | H-8 | C-7, C-8, C-9 |
| 1' | 100.2 | 4.33, br. d | H-2' | C-2', C-5', C-22 |
| 2' | 70.6 | 3.60, m | H-1', H-3' | C-1', C-3', C-4' |
| 3' | 73.7 | 3.23, m | H-2' | C-4' |
| 4' | 67.2 | 3.29, m | H-5' | C-3', C-5', C-6' |
| 5' | 77.5 | 3.00, m | H-4', H-6' | C-1', C-3', C-4', C-6' |
| 6' | 61.4 | 3.45, m, 3.67, m | H-5' | C-4', C-5' |

**Table S2. Comparison of the signature residues for amino acid selection by adenylation (A) domains of selected fungal PKS-NRPS (NRPS).**

| Protein (module) | Pos1a  (235) | Pos2  (236) | Pos3  (239) | Pos4  (278) | Pos5  (299) | Pos6  (301) | Pos7  (322) | Pos8  (330) | Pos9  (331) | Pos10  (517) | substrate |
| --- | --- | --- | --- | --- | --- | --- | --- | --- | --- | --- | --- |
| GrsA | **D** | A | W | T | I | A | A | I | C | **K** | L-Phe |
| CcsA | **D** | M | S | E | S | W | C | F | C | **K** | L-Phe |
| PsoA | **D** | A | Y | T | S | W | A | I | C | **K** | L-Phe |
| EqiS | **D** | F | E | S | H | W | N | I | A | **K** | D-Ser |
| Pks3 | **D** | L | L | M | T | W | W | I | V | **K** | D-Ser |
| ATEG_00325 | **D** | A | S | L | Q | W | A | I | M | **K** | L-Leu |
| MycA | **D** | L | W | G | T | W | C | V | G | **K** | L-Leu |
| ApdA | **D** | M | V | I | Y | W | C | A | A | **K** | L-Tyr |
| TenS | **D** | M | V | I | T | W | C | A | A | **K** | L-Tyr |
| CpaS (*A*. *flavus*) | **D** | M | A | L | T | W | S | A | C | **K** | L-Trp |
| CpaS (*A*. *oryzae*) | **D** | M | A | L | A | W | S | A | C | **K** | L-Trp |
| UcsA | **D** | V | W | F | S | W | I | I | P | **K** | 3-methylproline |
| BuaA | **D** | V | Q | H | K | W | V | V | I | **K** | 4-methylproline |
| HTS1.2(NRPS) | **D** | A | G | G | C | A | M | V | A | **K** | D-Ala |
| CssA(NRPS) | **D** | L | L | F | G | I | A | V | L | **K** | L-Ala |
| HTS1.3(NRPS) | **D** | L | L | F | G | I | S | V | L | **K** | L-Ala |
| EasA4 | **D** | L | L | V | V | A | G | I | L | **K** | L-Ala |
| EasA5 | **D** | I | A | I | L | V | A | I | L | **K** | L-Ala |
| EpiA | **D** | L | G | I | F | G | V | V | W | **K** | L-Ala |

Note: The 10 signature residues were based on the bacteria A domain as established previously [1]. Sequence numbering is according to A domain of EpiA as predicted by antiSMASH 6.0 [2] (corresponds to amino acid 2951 – 3354 of the full length EpiA) and were based on homology modelling with bacterial A domain as performed previously by Stachelhaus *et al* [3]. The signature residue table above is an expansion the table in this previous study [4].

■ REFERENCES

1. Stachelhaus T, Mootz HD, Marahiel MA. [The specificity-conferring code of adenylation domains in nonribosomal peptide synthetases.](https://pubmed.ncbi.nlm.nih.gov/10421756/) Chem Biol. 1999; 6(8):493-505.
2. Blin K, Shaw S, Kloosterman AM, Charlop-Powers Z, van Wezel GP, Medema MH, Weber T. AntiSMASH 6.0: improving cluster detection and comparison capabilities. Nucleic Acids Res. 2021; 49:W29-W35.
3. Chiang YM, Szewczyk E, Nayak T, Davidson AD, Sanchez JF, Lo HC, Ho WY, Simityan H, Kuo E, Praseuth A, Watanabe K, Oakley BR, Wang CCC. Molecular genetic mining of the *Aspergillus* secondary metabolome: discovery of the emericellamide biosynthetic pathway. Chem Biol. 2008; 15:527-532.
4. Li H, Gilchrist CLM, Lacey HJ, Crombie A, Vuong D, Pitt JI, Lacey E, Chooi YH, Piggott AM. Discovery and heterologous biosynthesis of the burnettramic acids: rare PKS-NRPS-derived bolaamphiphilic pyrrolizidinediones from an Australian fungus, *Aspergillus burnettii*. Org Lett. 2019; 21:1287-1291.


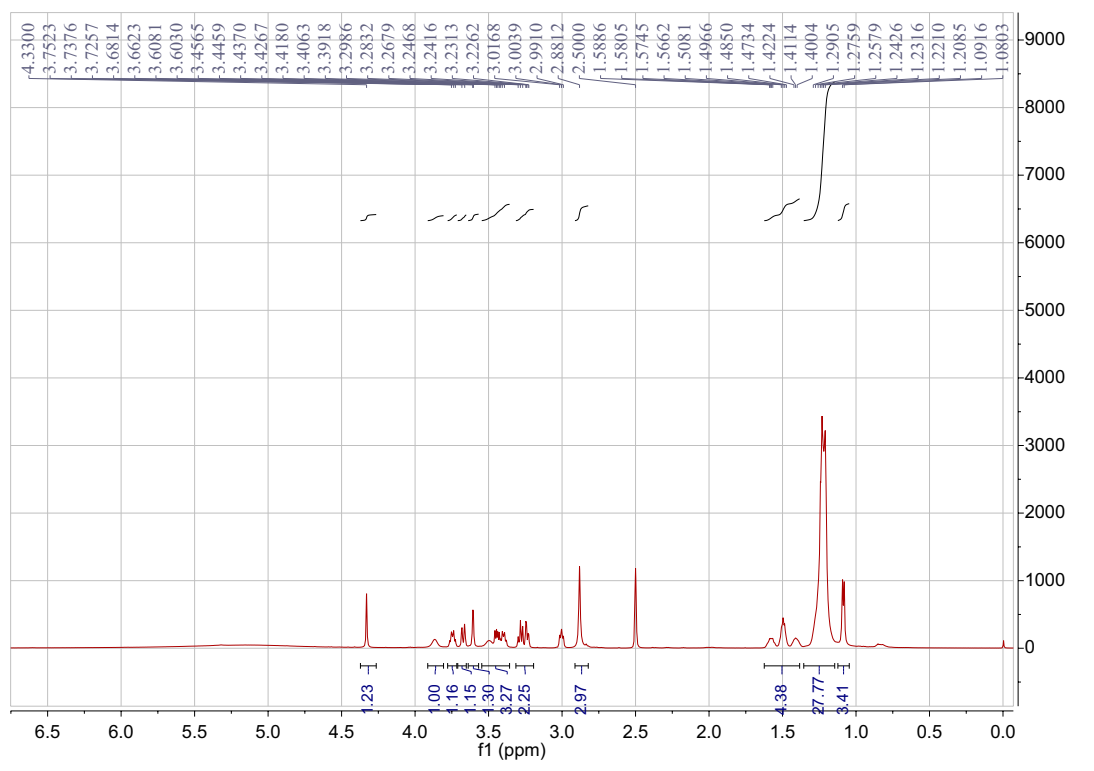


**Figure S1.** The ^1^H NMR specturm of compound **1**（in DMSO, 600MHz）


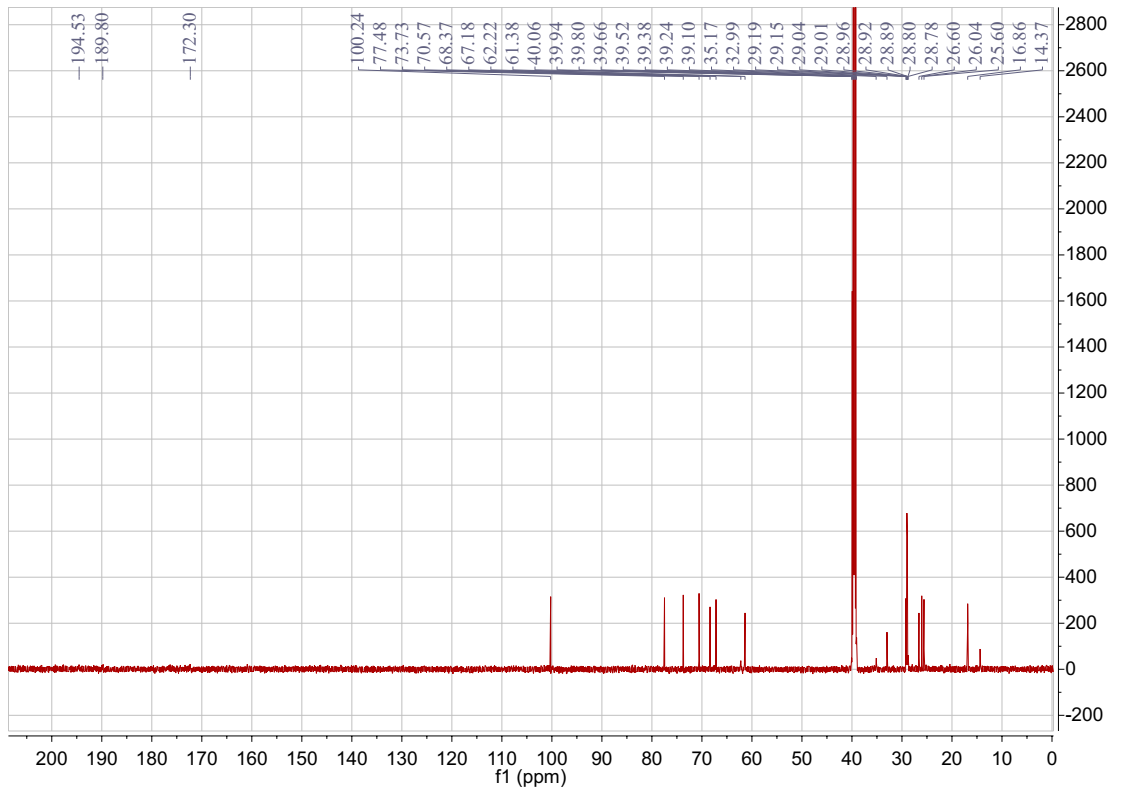


**Figure S2.** The ^13^C NMR spectrum of compound **1** (in DMSO, 151MHz)


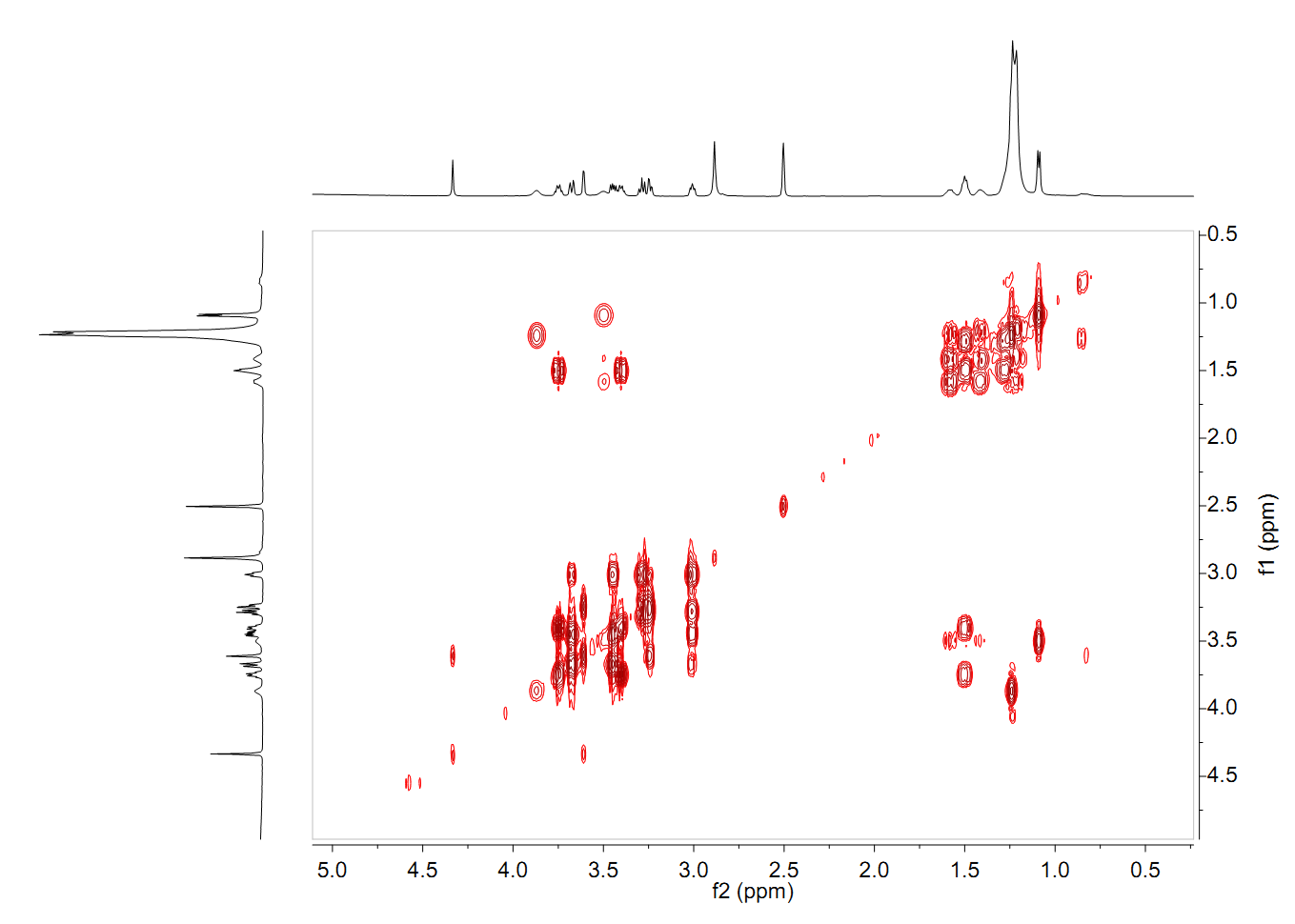


**Figure S3.** The ^1^H-^1^H COSY spectrum of compound **1** (in DMSO)


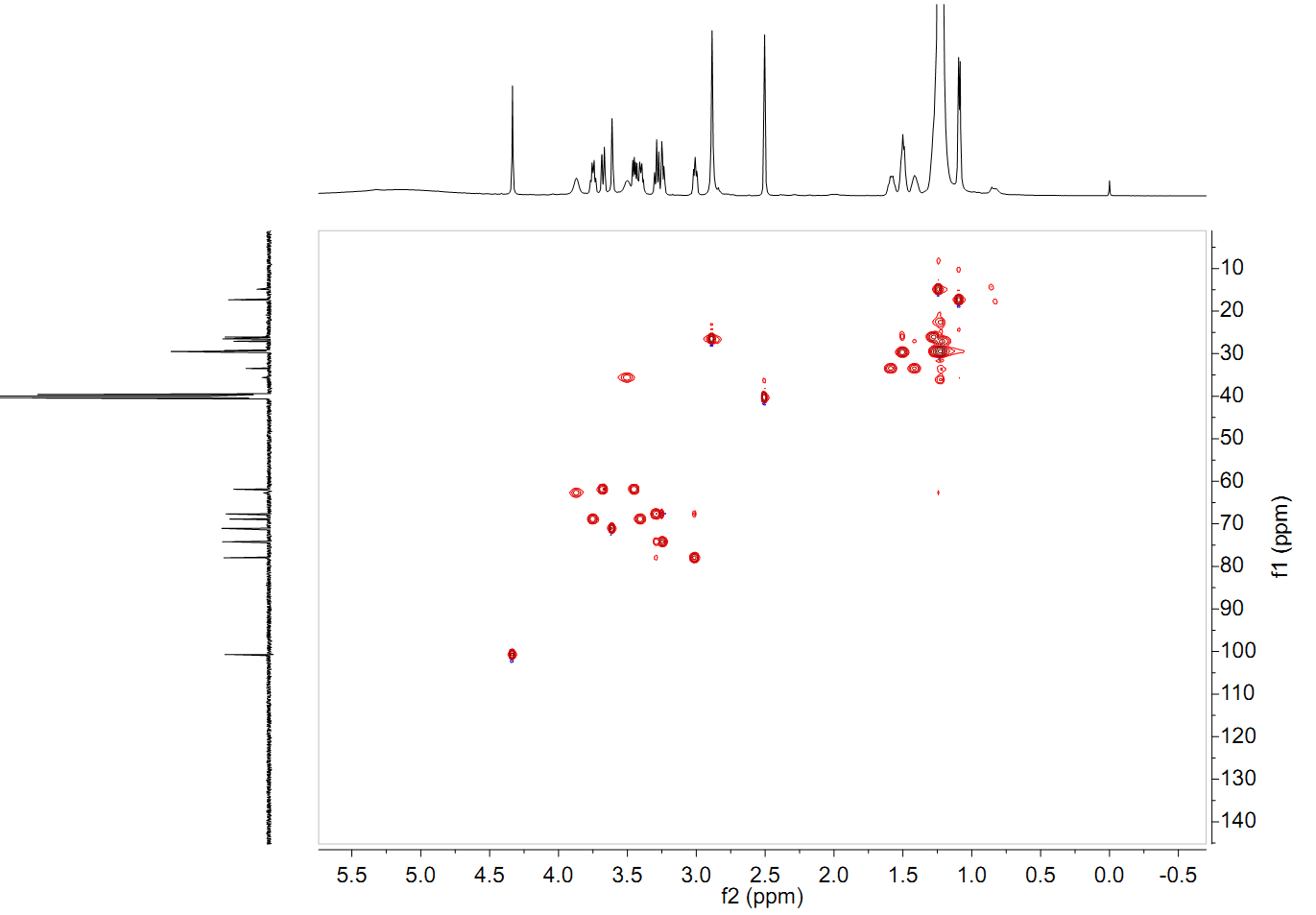


**Figure S4.** The HSQC spectrum of compound **1** (in DMSO)


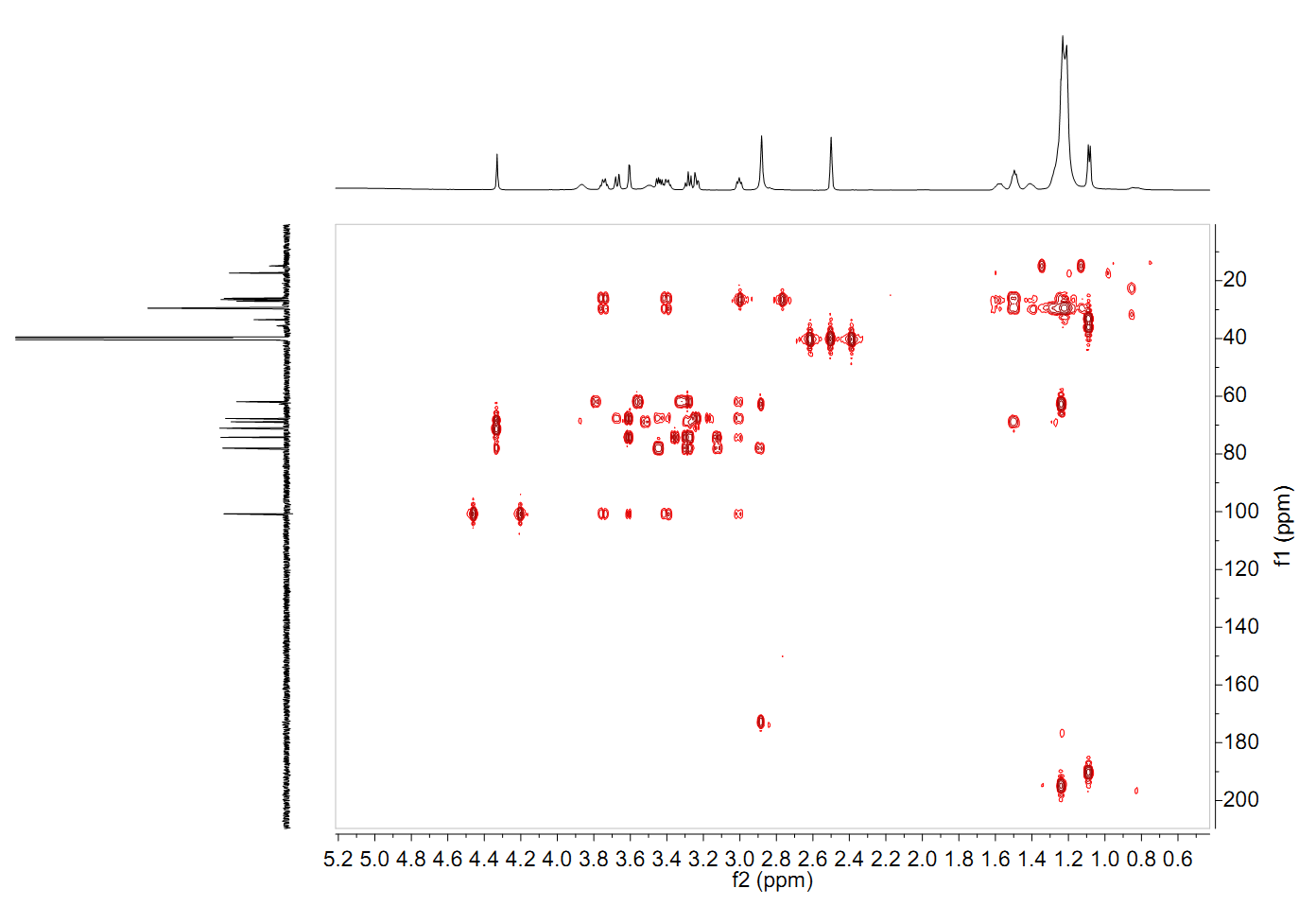


**Figure S5.** The HMBC spectrum of compound **1** (in DMSO)

**Figure S6.** HRESIMS spectrum of compound **1**

**Figure S7.** ESI MS/MS (-) spectrum of compound **1**
